# Beyond the Injury: A Case Report on Psychological Intervention During ACL Rehabilitation in a Professional Futsal Player

**DOI:** 10.3390/ijerph23010026

**Published:** 2025-12-23

**Authors:** Luis Miguel Ramos-Pastrana, Laura Gil-Caselles, Roberto Ruiz-Barquín, José María Giménez-Egido, Aurelio Olmedilla-Zafra

**Affiliations:** 1 Department of Psychology, University of Murcia, 30720 Murcia, Spain; luismiguel.ramos@um.es; 2 Research Group HUMSE (Human Movement and Sports Science), Faculty of Sports Sciences, University of Murcia, 30720 Murcia, Spain; josemaria.gimenez@um.es (J.M.G.-E.); olmedilla@um.es (A.O.-Z.); 3 Department of Education, Autonomous University of Madrid, 28049 Madrid, Spain; roberto.ruiz@uam.es; 4 Department of Personality, Evaluation, and Psychological Treatment, University of Murcia, 30720 Murcia, Spain

**Keywords:** anterior cruciate ligament, return to play, mental health, psychological intervention, rehabilitation, case report

## Abstract

**Highlights:**

**Public health relevance—How does this work relate to a public health issue?**

- ACL injuries represent a public health problem due to their high incidence in athletes, their psychological and functional impact, and the costs associated with rehabilita-tion and return to activity.
- The psychological consequences (anxiety, depression, fear of re-injury, etc.) are aspects of the problem that are rarely addressed and can compromise recovery and reintegra-tion into sport.

**Public health significance—Why is this work of significance to public health?**

- This study provides longitudinal evidence that psychological intervention sustainably improves key indicators of mental health, sleep, pain, and readiness to return to sport, thereby improving treatment compliance and rehabilitation outcomes.
- This reinforces the need to integrate psychological care into standard rehabilitation programs, with the potential to reduce recurrence, quitting sport and decrease psy-chological problems and the healthcare burden associated with ACL injuries.

**Public health implications—What are the key implications or messages for practitioners, policy makers and/or researchers in public health?**

- Continuous psychological monitoring throughout rehabilitation is important, sub-sequently integrating psychological indicators as a complementary criterion to phys-ical parameters in order to authorize a return to competition.
- Practitioners and the research community should promote multidisciplinary work and encourage broader studies that evaluate the impact of these interventions, with the aim of optimizing care and improving outcomes in relation to sports injuries.

**Abstract:**

Background: An anterior cruciate ligament (ACL) rupture is one of the most psychologically demanding injuries in professional sport. This study aimed to describe a structured psychological intervention conducted during the rehabilitation process following an ACL rupture in a professional female futsal player. Methods: A single-case longitudinal design was implemented with three phases (pre-test, intervention, post-test) across a 12-month rehabilitation period. Psychological assessment was conducted at four key points: initial evaluation, rehabilitation follow-up, medical discharge, and three- and six-month follow-ups. The battery included perfectionism (FMPS), anxiety (STAI), depression (BDI-II), mental health indicators (DASS-21, GHQ-12), sleep quality (PSQI), pain perception and catastrophizing (VAS, PCS), mood states (POMS), psychological readiness for return to play (PRIA-RS), and perceived intervention effectiveness. The program consisted of 15 individual sessions plus a follow-up, combining cognitive–behavioral therapy principles, mindfulness-based techniques (relaxation, body scan, visualization), cognitive restructuring, sleep hygiene, goal setting, problem-solving, and emotional expression strategies. Results: Progressive and sustained improvements were observed in mood states and pain catastrophizing, along with enhanced sleep quality, psychological readiness, and reintegration into competition. Improved overall mental health indicators were also observed, supporting adherence to rehabilitation and return-to-play confidence. Conclusions: This case highlights the relevance of structured psychological intervention as an integral component of injury rehabilitation in professional athletes with ACL rupture, supporting its inclusion in multidisciplinary care and future research to optimize recovery and prevent maladaptive outcomes.

## 1. Introduction

Athletes, like the general population, are vulnerable to mental health symptoms such as anxiety, depression, eating and sleep disorders, addictions, or suicidal ideation, often associated with the stress inherent in the sporting environment [1]. This same stress is also linked to an increased risk of injury and may influence both recovery and return to sport [2,3]. These psychological mechanisms are particularly relevant in athletes recovering from ACL injuries, who commonly experience fear of re-injury, pain-related catastrophizing, anxiety, and fluctuations in mood. The intervention components applied in this case, including CBT-based cognitive restructuring, mindfulness practices, goal-setting, sleep hygiene strategies, and guided visualization, directly target these vulnerabilities by reducing maladaptive thoughts, enhancing emotional regulation, and promoting a sense of control throughout the rehabilitation process.

Sports injuries, in addition to their physical impact, entail significant psychosocial consequences [4,5] and may trigger or exacerbate mental health issues [6,7]. They are also a frequent cause of career termination, often accompanied by identity loss or psychological distress [8]. In professional football, 62.9% of retired athletes cited injury as the primary reason, particularly injuries to the knee or ankle [9].

Although emotional responses to injury are usually temporary, some athletes display signs of poor adjustment, such as irrational fear, denial, irritability or obsessive thoughts about returning to play, which may require professional intervention [10]. Pain catastrophizing, kinesiophobia and low self-efficacy can hinder functional recovery and decrease athlete satisfaction [11,12,13]. Thus, psychological factors must be integrated into assessment protocols to prevent risky behaviors and guide safe return to sport [10,14].

Anterior cruciate ligament (ACL) rupture is among the most frequent and severe sports injuries, with higher prevalence in women and in sports such as football [15,16,17,18,19]. In futsal, the most common injuries involve knees and ankles, with ACL injuries standing out due to their severity [20,21,22].

Return after ACL reconstruction (ACLR) is typically based on physical criteria, often overlooking psychological readiness, even though physical and psychological recovery may not coincide [23,24]. Psychological factors, such as high levels of kinesiophobia and a lower psychological readiness to return to play (RTP), have been shown to hinder RTP, alter measurable knee biomechanics, and may increase the risk of re-injury [25]. In fact, delaying RTP by just a few weeks has been shown to significantly reduce reinjury risk [26].

Webster and Feller [27] proposed a continuum model of RTP that spans from initial participation to pre-injury performance levels, highlighting the importance of integrating psychological factors throughout the process. Psychological readiness to return encompasses perceptions of competence, identity and autonomy, as well as social support and expectation management [28,29]. This readiness fluctuates over time and may be affected by intrusive thoughts, worry, or external pressure [23].

Unrealistic expectations can lead to premature returns, heightened anxiety and increased risk of reinjury [23,30,31]. Conversely, early psychological readiness has been associated with better functional outcomes [32] and should be considered in clinical decision-making [33,34,35].

Key predictors of RTP include motivation, low kinesiophobia, self-efficacy, social support, and level of play [27,36,37]. The most common barriers are fear of reinjury, lack of confidence and personal obligations [38]. One year post-surgery, 15% of athletes discontinue sports participation, particularly women and those with prior surgical history [39].

Protective factors such as realistic expectations, coping strategies and social support can facilitate recovery [10]. Psychological interventions including visualization, relaxation, self-talk and goal setting have proven effective in reducing pain and anxiety and improving adherence to rehabilitation [40,41,42,43,44]. Studies involving injured athletes with ACL rupture have validated the effectiveness of visualization [45], mindfulness [46] and remote cognitive–behavioral programs [47], showing improvements in mood, self-efficacy and readiness to return. The use of psychological skills such as self-talk and goal setting has also enhanced adherence and engagement in home-based rehabilitation exercises [48,49]. However, due to the high risk of bias, the quality of evidence for these outcomes is very low [50].

Nonetheless, many athletes remain reluctant to address the psychological dimension of their injuries. Only 22.4% seek professional help, with stigma, lack of awareness and fear of losing sporting opportunities cited as the main barriers [51]. Support from coaches and teammates, alongside the normalization of psychological care within sporting contexts, are key to encouraging help-seeking behavior [23,52,53].

Traditionally, medical approaches have prioritized physical and biomechanical factors, underestimating the influence of psychological aspects, despite over five decades of research demonstrating their critical role in injury prevention, rehabilitation and return to sport [54]. Therefore, it is essential to incorporate psychological interventions to promote comprehensive and sustainable recovery.

The aim of this study is to present a psychological intervention implemented during the rehabilitation of an ACL rupture in a professional female futsal player, analyzing its effects on variables such as mood, pain perception, sleep, mental health, and psychological readiness to return to sport, and assessing whether such readiness occurred at an appropriate time.

## 2. Materials and Methods

### 2.1. Design

This study adopted an observational, longitudinal single-case design (*n* = 1) comprising three distinct phases (pre-test, intervention, and post-test), following the classification proposed by Ato et al. [55]. The aim was to provide an in-depth description of the psychological course of a professional futsal player during ACL rehabilitation while a structured psychological intervention was integrated into standard medical and physiotherapy care. Given its uncontrolled A–B nature, this design does not allow causal inferences about the effects of the intervention and can only describe a temporal sequence of changes within a complex rehabilitation process. The process unfolded over the course of one year, from the time of injury to the athlete’s return to competition. The injury occurred on 1 October, with rehabilitation commencing on 11 October. Surgical intervention was carried out on 14 November. Psychological intervention began on 13 December and continued until 30 June. The futsal player was medically discharged on 1 August and played her first official match on 12 September.

This methodological approach was selected because ACL rehabilitation in elite athletes requires highly individualized and continuous monitoring, making single-case methodology particularly appropriate for capturing psychological and clinical changes within the same individual. More complex variants such as A–B–A or multiple-baseline designs were not feasible in this real-world clinical context, as withdrawing or interrupting the intervention would have been ethically inappropriate and incompatible with the athlete’s rehabilitation schedule. Likewise, establishing multiple baselines across participants was not possible because only one athlete met the inclusion criteria during the intervention period.

### 2.2. Participant

The participant was a 22-year-old professional futsal player competing in the First Division of the Spanish League. She had an extensive sporting background, with 19 years of practice in futsal, and had represented the Spanish national team, winning two European Championships and several national titles with a regional football federation. She was also enrolled in a bachelor’s degree in physical Activity and Sport Sciences.

The futsal player had a history of injuries to her ankles and knees and did not receive psychological treatment for these injuries. Specifically, she had sustained a meniscus tear in her right knee (with an estimated recovery time of three months), two sprains in her left ankle—each requiring an estimated recovery period of about four months—and a fracture in her right knee, with a recovery time of three to four months. On 1 October 2022, during an official league match, she sustained a rupture of the anterior cruciate ligament (ACL) in her left knee due to poor footing following a push by an opponent. She underwent surgery on 14 November 2022, involving ACL reconstruction using a hamstring tendon graft. The estimated recovery period ranged from eight to nine months.

### 2.3. Instruments

A semi-structured interview was conducted to collect information on sociodemographic variables and the context of the sports injury, based on the work of Olmedilla-Caballero et al. [56] The interview addressed aspects related to the ACL injury, including sporting and psychosocial background, circumstances surrounding the injury, and immediate consequences. Additionally, following the methodology of Palmi et al. [46], the interview incorporated the assessment of perceived emotional variables, coach demands, personal motivation, self-demand, task focus, and psychological coping resources (Supplementary File S1).

Among the questionnaires used was the Frost Multidimensional Perfectionism Scale (FMPS) [57], in the Spanish version adapted by Carrasco et al. [58] consisting of 35 items rated on a five-point Likert scale. It assesses four main dimensions: fear of mistakes (11 items; α = 0.88), parental influences (9 items; α = 0.90), achievement expectation (9 items; α = 0.87), and organization (6 items; α = 0.89). Two higher-order factors can be derived: functional perfectionism (achievement expectation and organization) and dysfunctional perfectionism (parental influences and fear of mistakes).

Anxiety was assessed using the State-Trait Anxiety Inventory (STAI) Spanish version, ref. [59] comprising 40 items across two subscales: State Anxiety (α = 0.92) and Trait Anxiety (α = 0.89). Items are scored on a four-point Likert scale (0 = not at all; 3 = very much so), distinguishing between situational and dispositional anxiety.

The Beck Depression Inventory-II (BDI-II) [60] was also administered in the Spanish adaptation by Sanz et al. [61]. It comprises 21 items on a four-point Likert scale, with items 16 and 18 having seven response categories. Total scores range from 0 to 63, allowing classification into minimal (0–13), mild (14–19), moderate (20–28), and severe (29–63) depression. Internal consistency is high (α > 0.85).

To provide a more specific assessment of stress, anxiety and depression levels, the DASS-21 Scale, ref. [62] in the Spanish version validated by Fonseca-Pedrero et al. [63] was used. The questionnaire comprises 21 items across three subscales—depression (α = 0.80), anxiety (α = 0.73), and stress (α = 0.81)—with an overall reliability of α = 0.90.

Perceived general health was measured using the General Health Questionnaire (GHQ-12) [64], with the Spanish adaptation by Sánchez-López and Dresch [65]. It includes 12 items scored on a four-point Likert scale, yielding a total score ranging from 0 to 36, where higher scores indicate greater psychological distress. Internal consistency was α = 0.76.

Sleep quality was assessed using the Pittsburgh Sleep Quality Index (PSQI), ref. [66] Spanish version by Royuela and Fernández [67]. It consists of 19 self-reported items and 5 observer items covering seven components of sleep. Each component is rated from 0 to 3, resulting in a global score ranging from 0 to 21, with a cut-off score of 5. Reliability (α) ranges from 0.67 to 0.81 depending on the population.

Cognitive-emotional response to pain was evaluated using the Pain Catastrophizing Scale (PCS), ref. [68] adapted for athletic populations by Olmedilla et al. [69] It consists of 13 items distributed across three dimensions: rumination (α = 0.735), helplessness (α = 0.737), and magnification (α = 0.618), with a total reliability of α = 0.818.

The Visual Analog Scale (VAS) [70] was also used, a unidimensional tool for quantifying perceived pain intensity on a scale from 0 (no pain) to 10 (unbearable pain).

Emotional states were assessed using the Profile of Mood States (POMS) [71], Spanish version by Fuentes et al. [72]. This 29-item Likert-type scale measures five affective dimensions: tension (α = 0.83), depression (α = 0.78), anger (α = 0.85), vigor (α = 0.83), and fatigue (α = 00.82). The scores for each factor were standardized on a 0–100 scale using a simple rule-of-three conversion. For example, the maximum score for the tension factor is 24, which corresponds to a value of 100 on the transformed scale.

Regarding readiness to return to sport, the PRIA-RS Questionnaire was applied [73]. It assesses the injured athlete’s self-perceived readiness to return to training and competition. The tool includes 10 items with various response formats, and the total score reflects different levels of predisposition for return. Internal reliability in injured football populations is α = 0.81 [74].

Perceived effectiveness of the psychological intervention was assessed using a questionnaire previously employed in research, refs. [56,75] which includes items on satisfaction, usefulness, transferability, and techniques learned (Supplementary File S2).

Analysis of the return to competition. The player’s first matches were recorded in a similar way to the recording method used by Palmi et al. [46]. The match, results, performance rating (Likert scale from 0 = very low to 10 = very high), emotional state and the player’s observations were taken into account (Supplementary File S3).

### 2.4. Procedure

Prior to the start of the study, the participant provided written informed consent, and all procedures were conducted in accordance with ethical standards, ensuring that the rights and confidentiality of the participant were fully protected.

The athlete was contacted through a sports polyclinic and the psychological support service of a regional football federation. Psychological assessment was online and conducted at four key time points: initial evaluation, follow-up during rehabilitation, medical discharge, and follow-ups at three- and six-month post-discharge. During the initial evaluation, the following instruments were administered: VAS, FMPS, STAI, POMS, BDI-II, PCS, and PSQI. During the follow-up phase, the VAS, PCS, POMS, DASS-21, GHQ-12 and PSQI were re-administered. At the time of medical discharge, the DASS-21, GHQ-12, PSQI and PRIA-RS were used, and the subsequent follow-ups included repeated assessments using the POMS, DASS-21, GHQ-12 and PSQI. The battery of tools employed adhered to the “Psycholight” protocol developed by Olmedilla and García-Mas [76].

The intervention was grounded in cognitive–behavioral therapy (CBT) and its four core principles [77], with the aim of promoting psychological well-being, enhancing treatment adherence, increasing self-efficacy, and optimizing recovery. Thus, working on goals and expectations was the starting point. Goals were formulated for the short, medium, and long term, and were explicit, measurable, achievable, and positively framed. Both performance goals (e.g., completing a specific number of rehabilitation exercises) and outcome goals (e.g., walking unaided) were included, with prioritization of performance goals due to these goals depended more on the player [78,79,80].

Subsequently, the introduction of attention focuses was intended to enhance the player’s ability to direct her attention both to bodily sensations and to her thoughts [81], which facilitated the subsequent implementation of techniques such as relaxation, visualization, and cognitive restructuring.

Relaxation strategies included diaphragmatic breathing [82] and the “Body Scan” technique from the Mindfulness approach [83], aimed at emotional regulation, pain management, and enhancing general well-being. These techniques have demonstrated efficacy in injured athletes in reducing anxiety and stress [84].

Relaxation was also incorporated to improve sleep quality, as this directly influences mood and the level of energy needed for proper rehabilitation. Therefore, it was complemented with sleep hygiene and stimulus control strategies [85], such as avoiding screen use before bedtime, maintaining consistent sleep schedules, and limiting long naps, in order to re-establish a positive association between bed and rest.

Active coping skills and problem-solving abilities were also trained to minimize the impact of concerns about rest and well-being, following the guidelines of Olivares et al. [86]. This training involved defining the problem, setting clear goals, generating alternative solutions, planning steps sequentially, and verifying outcomes. Emphasis was placed on focusing on controllable aspects, personal responsibility, and the cognitive reappraisal of the injury event as a challenge rather than a threat. During the initial interview, the athlete also reported difficulty expressing emotions and described a sense of emotional numbness. To address this, a writing technique was implemented based on the work of Mankad and Gordon [87], focused on the “letter to the injury”. The athlete was invited to name her injury and write about her rehabilitation experience, including unspoken thoughts, emotions, and expectations. This activity served as a cognitive restructuring tool, the effects of which were evaluated at the end of the intervention.

The previous coping strategies served as a prelude to more in-depth cognitive restructuring work, especially with regard to generating alternatives and identifying consequences. Cognitive restructuring was approached through the use of A-B-C thought records (activating event, belief, emotional/behavioral consequence), which enabled the identification and modification of automatic negative thoughts [88]. Additional techniques included thought-stopping, behavioral and cognitive distraction strategies, and the so-called “worry time”, in which the athlete dedicated a specific time of day to process recurring concerns.

Finally, visualization was used at various stages of the process [89]. Initially, it served as tool for relaxation and well-being. However, it was decided to delve deeper into more advanced stages, as this required adequate management of the level of activation and sufficient cognitive control. In addition, it was used as an imagined practice to accompany more complex exercises in the physical and sports rehabilitation process, with the aim of strengthening the athlete’s confidence and reducing the fear of reinjury. The effectiveness of this technique in athletic populations has been supported by recent studies [90,91].

The intervention was structured into 15 sessions (Table 1), with a follow-up session held three months after medical discharge. Sessions lasted approximately 50 min and were primarily conducted in person at a room in the Faculty of Psychology at a university, although some were delivered online due to logistical reasons (academic, travel, work or rehabilitation constraints). The session format typically included: 10 min for reviewing updates and homework, 10 min of theoretical explanation, 25 min of guided practice, and a final 5 min to set new tasks which consisted of putting into practice the techniques and tools worked on in the session.

### 2.5. Statistical Analysis

Given the nature of this single-case A–B design, the analysis focused on intra-individual changes over time using descriptive statistics and visual inspection of score trajectories across the different assessment points. This approach is consistent with recommendations for clinical single-case research when the number of observations per phase is limited.

No inferential statistical tests were applied, as the study was not designed to establish causal effects. Although SCED statistical indices (e.g., Tau-U, Nonoverlap of All Pairs) can be used in single-case methodology, they were not appropriate in this context due to the small number of repeated measures and the clinical timing of assessments, which did not allow for stable baselines or sufficient data points per phase. Therefore, results are interpreted descriptively, focusing on clinically meaningful changes and temporal patterns.

## 3. Results

The results obtained by the player in the evaluation instruments are presented below (Table 2). Based on the cut-off points of the BDI-II, the player manifested mild depressive symptomatology. Her score for Anxiety-Trait is at the 50th percentile, which means that player scores above the middle of her reference group. On the other hand, their score for Anxiety-State is at the 35th percentile, leaving 35% of their reference group below. Finally, the player scored above the 75th percentile on most subscales and achieved functional perfectionism above dysfunctional perfectionism. For both instruments (STAI and FMPS), the norms of the instruments based on the general population were used.

**Figure 1:**
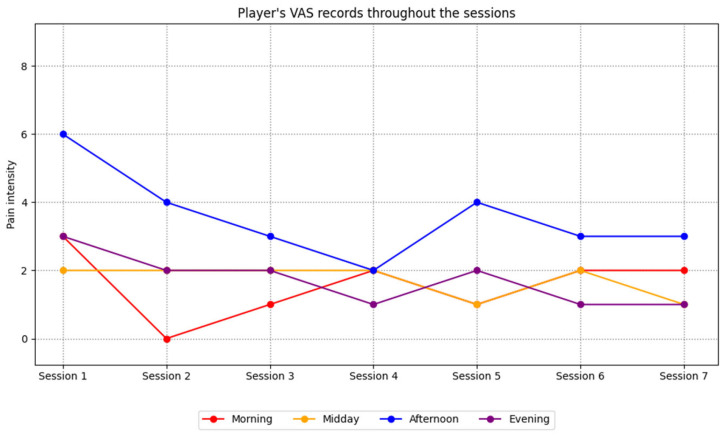
Player’s EVA records throughout the sessions.

The VAS records results are summarized in Figure 1. Player perception of pain decreased during the intervention process, especially in the afternoon and evening

Table 3 shows the scores obtained in the assessment of pain catastrophism (PCS). The results indicate that there was a significant reduction in all catastrophism factors.

**Figure 2:**
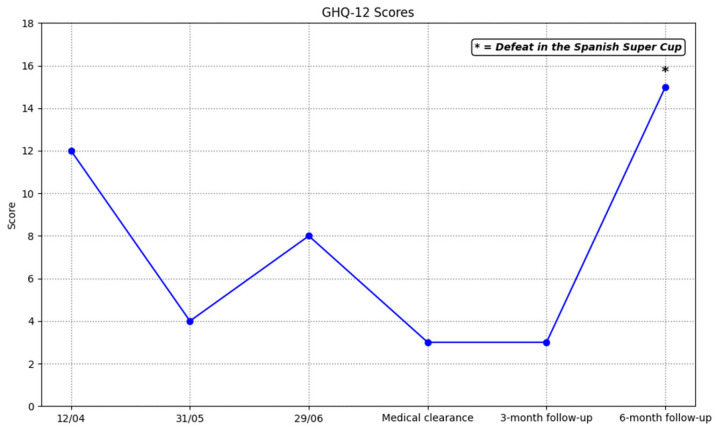
Evolution of scores on the GHQ-12.

The GHQ-12 scores are presented in Figure 2, including assessment at medical clearance and follow-up at 3 and 6 months after medical clearance. Scores fell, indicating better mental health. However, at the 6-month follow-up, there was a score increase coinciding with a defeat in the Spanish Super Cup.

**Figure 3:**
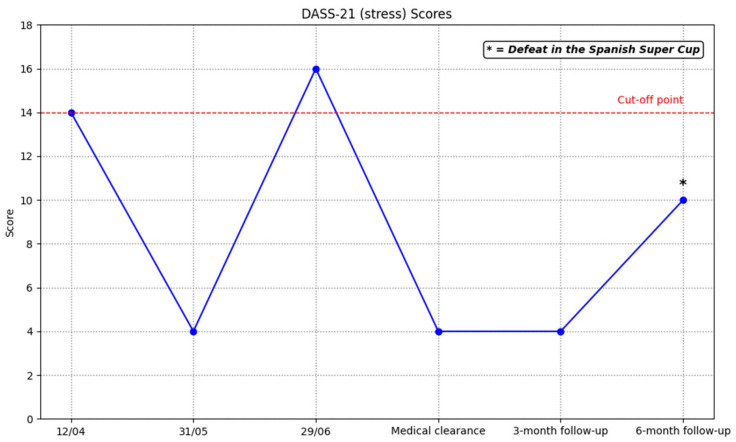
Evolution of scores on the DASS-21 (stress).

Figure 3 shows the player’s evolution in the DASS-21 Stress scale scores. According to the cut-off points established by Lovibond and Lovibond [62] in most measurements the player was at normal stress levels.

At the 3- and 6-month follow-ups after medical clearance, scores on all scales remained within normal ranges (Table 4).

**Figure 4:**
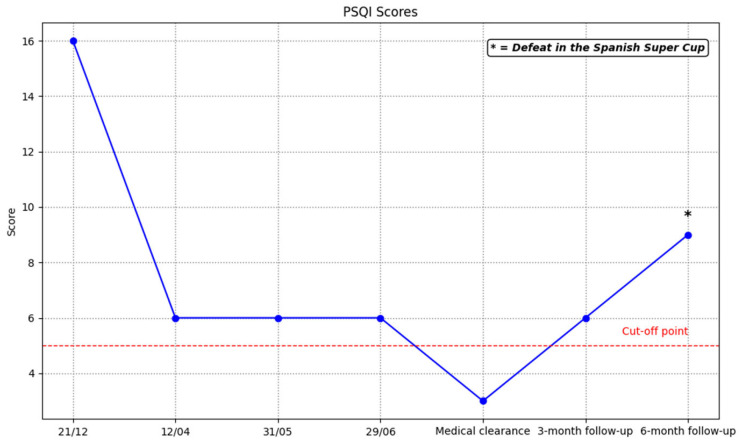
Scores obtained on the PSQI throughout the intervention.

The PSQI results (Figure 4) show a decrease in sleep quality scores, indicating an improvement. However, values above 5 still indicate suboptimal sleep.

**Figure 5:**
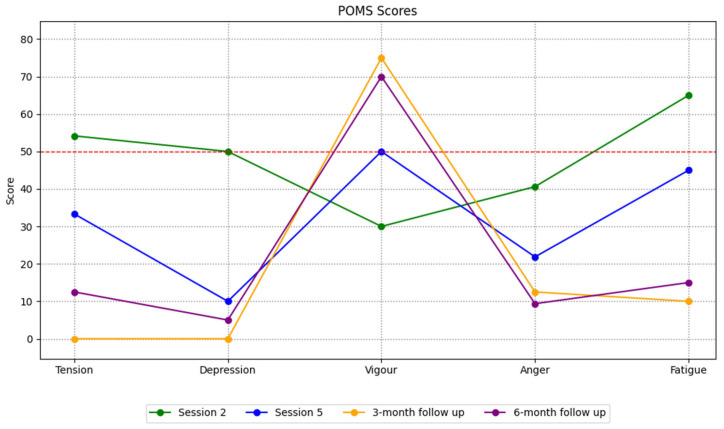
Scores obtained on the POMS throughout the intervention.

Figure 5 shows the player’s mood profile in the initial assessment (Session 2) and the profile obtained after Session 5 of psychological intervention. The scores have been converted to a scale ranging from 0 to 100 points, with 50 representing the midpoint. As demonstrated in Session 2, the player is characterized by the presence of negative factors close to or above the central score, while the Vigor factor stands out as scoring lower than all the others. Conversely, in Session 5, the desirable profile delineated by Morgan [92] is observed, wherein the Vigor factor is elevated, while the remaining factors fall below the midpoint. Compared to the previous profiles, the profiles found after medical clearance indicate an increase in the Vigor factor and a decrease in the other negative factors.

Table 5 shows the cut-off points of the PRIA-RS and the player’s score. The results show the player was confident about her injury, her knee’s functionality, and that she was not afraid of another injury. The player perceived herself to be psychologically prepared for her return to sport.

Finally, with regard to the evaluation of the psychological intervention, on a scale of 10 the participant assigned a rating of 9 to her level of satisfaction with the psychological intervention, 10 to its usefulness in the rehabilitation process, return to sport, training and competition, and 9 to its usefulness in other areas. In terms of satisfaction and the effectiveness of the techniques and strategies learned, the participant assigned high ratings to all of them, with the lowest rating being 8 for cognitive restructuring. In terms of qualitative analysis, the participant noted that visualization helped her to imagine things she could not to do at the time and thereby instilling motivation. The work on sleep hygiene was significant in that it facilitated the establishment of new habits, thereby enhancing performance levels. Furthermore, the player emphasized the significance of relaxation as she considers herself to be a very nervous person and tends to stress about everything straight away.

## 4. Discussion

The objective of this research was to present the psychological intervention during the ACL rehabilitation process of a professional futsal player. In consideration of the results obtained and the player’s self-assessment of the psychological intervention, it can be concluded that in this single case, we observed improvements in mood, sleep and psychological readiness that coincided with the implementation of the psychological intervention within the rehabilitation program. This finding aligns with the conclusions of previous reviews, which have reported that the implementation of techniques and strategies such as relaxation, visualization, goal setting and written emotional expression can promote positive changes in mood, pain management and adherence to rehabilitation [40,41].

Firstly, mild depressive symptoms were observed in the initial evaluation. These results align with the systematic review by Piussi et al. [93] which found that athletes who had suffered ACL injuries might experience depressive symptoms, especially in the first six weeks after surgery, and more common in professionals than amateurs. The player also exhibited a higher level of functional perfectionism than dysfunctional perfectionism. This profile is characterized by high levels of organization, high personal standards, and achievement orientation. This trait probably facilitated greater adherence to rehabilitation exercises and psychological treatment, as the athlete strictly followed the guidelines and strove to complete each session correctly. In addition, the focus on improvement and achievement may have fostered a more positive interpretation of the process, such as having more tools and being mentally stronger.

In relation to previous studies, Olmedilla et al. [94] also reported that high adaptive or functional perfectionism was linked to a reduction in anxiety, stress and depression symptoms in a group of female football players. In contrast, those with high maladaptive perfectionism had increased symptoms and injury risk. Similar results were also found by González-Hernández et al. [95], the authors found that functional perfectionism was linked to reduced injury history and catastrophizing. In contrast, dysfunctional perfectionism was linked to increased injury history, catastrophizing, anxiety sensitivity and previous injury severity.

Results show that pain perception and catastrophizing decrease as intervention continues. First, the 2–3 point reduction (especially in the afternoon and evening) in pain perception measured using the VAS exceeded the estimated MCID of 0.9–1 point (scale adjusted to 10 points) for ACL patients described in previous studies [96,97]. Similar findings have been seen in other interventions [98]. Brewer et al. [99] developed a multimedia programmed for athletes with ACL injuries. The program provides information before surgery and techniques like goal setting, visualization, modeling, and positive self-talk. The intervention group showed greater preoperative confidence, less postoperative pain and kinesiophobia, and greater use and perceived usefulness of patient education materials. Mohammed et al. [42] conducted a mindfulness intervention with athletes with serious injuries. The intervention consisted of exercises like breathing meditation or ‘Body Scan’ to increase awareness of thoughts, bodily sensations and emotions, with an attitude of curiosity, openness and acceptance. The authors reported a notable reduction in anxiety and stress, as well as improved pain tolerance. Consequently, athletes who have sustained injuries may find this form of psychological intervention advantageous in terms of mitigating salient aspects associated with discomfort during their rehabilitation.

This study also observed that the total catastrophizing score decreased by 75.8% (from 33 to 8). Research has indicated a correlation between catastrophizing and the mood of injured football players, with greater catastrophizing being associated with more negative mood [100]. Conversely, athletes with severe catastrophizing are more likely not to return to a similar level of sport, and improvement in kinesiophobia after knee surgery is less likely with a higher frequency of preoperative perceived pain [26].

Studies have shown that mental health symptoms can reduce the effectiveness of physiotherapy interventions in reducing pain catastrophizing [101]. In this case, the player’s mental health improved, as shown by a decrease in GHQ-12 scores and the depression, anxiety and stress scales (DASS-21) remained within normal ranges at follow-ups at three and six months post-medical discharge. These findings are relevant because athletes with ACL injuries tend to perform fewer rehabilitation exercises at home when they are stressed or in a negative mood, which can affect adherence and rehabilitation outcomes [48]. In addition, those with high levels of stress or anxiety could amplify their perception of pain and concerns about recovery. Previous studies have also shown an association between serious injuries and psychological vulnerability in professional footballers. Gouttebarge et al. [102] pointed out that this type of injury is related to symptoms of distress, anxiety, sleep disturbances, and behavioral problems. Similarly, Kiliç et al. [103] reported a higher risk of mental health problems in players with a history of serious injuries.

From a dynamic perspective, psychological recovery after a sports injury may fluctuate depending on exposure to situational stressors, without this implying a sustained loss of the benefits of the intervention. The rebound observed six months after medical discharge in GHQ-12 scale coincides with a defeat in an important competition. This is a clear example of how exposure to acute stressors associated with returning to sports activity, such as increased competitive demands, pressure to perform, and fear of re-injury, can influence the results observed. It is also important to consider that the changes observed over time cannot be attributed exclusively to the psychological intervention, as external factors such as advances in physical rehabilitation or personal circumstances may also have contributed to both the initial improvement and the subsequent evolution of mental health.

Sleep disorders negatively affect performance, injury risk, recovery, and overall health in athletes [104]. In the present study, psychological intervention improved the player’s sleep quality, as reflected in lower PSQI scores. Various studies highlight the high prevalence of sleep dysfunction in footballers, especially in women, and its association with mental health problems [105,106]. Similarly, Khalladi et al. [107] emphasizes the role of sleep in the ACL rehabilitation process, noting that better sleep quality and lower anxiety levels predict a higher probability of meeting the criteria for return to play (RTP), as well as greater adherence to treatment. In this regard, effective sleep hygiene intervention could contribute positively to psychological well-being and functional recovery in injured athletes. However, it is important to acknowledge that improvements in sleep and mental health may also have been influenced by co-interventions inherent to the rehabilitation process, such as physiotherapy load, medical progression milestones, training demands, and the competition schedule. These external factors represent potential confounders that should be considered when interpreting the observed outcomes.

The results show that psychological intervention could improve mood. Initially, player presented an emotional profile dominated by negative factors. This changed over time, becoming an iceberg-shaped profile indicating good mental health [92]. Similar findings were reported with a decrease in negative dimensions of the POMS and an increase in Vigor after a mindfulness-based intervention with an ACL-injured footballer [46]. Podlog et al. [108] also found significant improvements in the experimental group compared to the control group after a cognitive–behavioral intervention, with a greater presence of positive emotions, less negative effect and greater vitality. This evidence supports the role of psychological interventions in improving the emotional well-being of athletes experiencing serious injuries.

Psychological readiness is essential for a safe return to sport after ACL injury. It has been reported that 64.7% of athletes who did not return attributed the cause to psychological factors, mainly fear of re-injury (76.7%), followed by lack of confidence in the knee (14.8%), depression (5.6%) and low motivation (2.5%) [109]. Recent studies have also observed poorer physical performance in footballers who do not feel psychologically prepared for return [110,111]. In the present study, the PRIA-RS assessment showed that the player was psychologically prepared, with confidence in the rehabilitation and functionality of the knee, absence of fear and good mood, findings consistent with Gómez-Espejo et al. [112], who linked a good psychological disposition with healthy emotional profiles.

Considering that the player had a history of injuries, including meniscus surgery during rehabilitation, these findings are related to the study by Xiao et al. [113]. Although they found no differences in self-reported knee function between athletes who returned to play and those who did not, they did find greater psychological readiness, self-efficacy, and less kinesiophobia in those who returned. Some authors also indicate that better psychological preparation and less fear are associated with a successful return without relapse [33,34,114]. However, recent studies warn that high psychological preparedness and self-efficacy may increase the risk of a new ACL rupture in the two years following reconstruction [30,31]. This could be explained by rapid progression in rehabilitation, which ignores biological recovery and key phases of readaptation, prematurely exposing the athlete to risks. In this case described, the athlete obtained a psychological readiness score just above the established threshold, reflecting an adequate level with no signs of overconfidence. Furthermore, the return to competition took place only after medical clearance and in accordance with the established timetable, ruling out a premature return associated with the risk of relapse. Finally, the six months of clinical follow-up without recurrence or signs of a hasty return confirms that the level of psychological readiness did not translate into reckless behavior or a discrepancy between perceived self-efficacy and actual physical condition. Therefore, it is crucial not only to encourage adequate psychological readiness, but also to closely monitor those with high readiness who meet the return criteria early in order to minimize potential complications.

## 5. Limitations and Future Directions

As this is a single-case A–B study, this research has important limitations inherent to this design. Given its uncontrolled nature, the changes observed in psychological and clinical variables cannot be attributed solely to the psychological intervention, as they are inevitably intertwined with the natural healing process after ACL reconstruction, the effects of surgery and physiotherapy, the passage of time, the athlete’s inherent resilience, and possible placebo or attention effects derived from receiving close professional support. Therefore, this report should be interpreted as a descriptive and exploratory case study rather than as an evaluation of intervention efficacy.

The intervention was carried out at an individual and professional level, specifically adapted to the athlete’s needs, which limits standardization and generates possible biases related to the professional in charge and the difficulty in controlling external variables such as social support, the rehabilitation environment, or unforeseen events such as meniscus surgery during ACL recovery. The use of several self-report measures on the same participant at multiple points in time can cause effects such as respondent fatigue, measurement reactivity, and shared method variance. The observed changes could be artifacts of repeated testing or the athlete’s desire to please the therapist. As future lines of research, it is proposed to complement self-reports with multi-method measures (e.g., observational or physiological) to improve ecological validity and reduce potential biases and develop studies with larger groups and more robust single-case designs (e.g., multiple-baseline or A–B–A structures) to compare different techniques and numbers of sessions, in order to standardize and streamline protocols.

Another limitation was the limited interdisciplinary work with physiotherapists, rehabilitation specialists, physical trainers, and coaches. Integrating psychological intervention into the overall rehabilitation program from a truly multidisciplinary approach could promote a more comprehensive and potentially more effective recovery.

On the other hand, one of the strengths of the study was the follow-up at 3 and 6 months after medical discharge, which allowed the sustainability of the observed changes to be evaluated over time. Despite the limitations, case studies are essential for highlighting the work of sports psychologists and providing practical, hypothesis-generating insights for clinical application in this field.

## 6. Conclusions

Within the constraints of a single-case design, this study describes how an individually tailored psychological intervention was integrated into ACL rehabilitation in a professional futsal player and how this process was accompanied by clinically relevant changes in psychological and clinical variables. These findings should be interpreted as exploratory and hypothesis-generating, rather than as evidence of intervention efficacy.

Based on this single case, several tentative conclusions can be drawn. Firstly, early screening of relevant psychological variables, such as mood, pain, or stress, appears essential for identifying athletes at greater risk of poor adherence or adverse outcomes during rehabilitation. Secondly, it is crucial to ensure that the athlete not only meets the physical and medical criteria for RTP but also has the appropriate psychological readiness. Excessively high levels of self-efficacy and readiness can, paradoxically, lead to premature return and increase the risk of relapse.

Likewise, the positive changes observed in mood, pain perception, catastrophizing, mental health, sleep quality, and psychological readiness for RTP in this case are consistent with the potential usefulness of integrating structured psychological support into ACL rehabilitation, although these changes cannot be causally attributed to the intervention alone. Finally, further research is recommended to validate the systematic incorporation of psychological interventions into conventional ACL rehabilitation programs, using controlled single-case and group designs and promoting a multidisciplinary approach that integrates close collaboration with doctors, physiotherapists, and rehabilitation specialists.

## Figures and Tables

**Table 1:** Session structure table.

Session	Technique Developed
1	Informed consent, initial interview, and explanation of the intervention program.
2	Initial evaluation
3	Goal setting and expectations
4	Attentional focus and relaxation techniques
5–7	Sleep hygiene, emotional regulation (letter to injury), problem solving and decision-making
8–10	Cognitive restructuring
11–14	Visualization
15	Follow-up and evaluation of psychological intervention

**Table 2:** Scores obtained in the initial assessment.

Questionnaire	Direct Score	Interpretation
Depression (BDI-II)	14	Mild depressive symptomatology
Anxiety (STAI)	Direct score	Percentile
Trait	24	50
State	17	35
Perfectionism (FMPS)	Direct score (% of maximum possible score)	Percentile
Fear of mistakes	34 (61.8)	>75
Parental influences	21 (46.7)	>75
Achievement expectation	31 (68.9)	>75
Organization	20 (66.7)	<75
Total Score	106 (60.6)	>75
Dysfunctional perfectionism	55 (55)	-
Functional perfectionism	51 (68)	-

**Table 3:** PCS scores throughout the intervention.

Date	Rumination	Helplessness	Magnification	Total Score
21 December	14	12	7	33
12 April	8	11	8	27
31 May	6	8	3	17
29 June	4	3	1	8

**Table 4:** Scores obtained on DASS-21 (stress) in follow-ups after medical clearance.

Scale	3-Month Follow-Up	Severity	6-Month Follow-Up	Severity
Depression	8	Normal	8	Normal
Anxiety	4	Normal	0	Normal
Stress	4	Normal	10	Normal

**Table 5:** Psychological readiness for the RTP at the time of medical discharge.

Cut-off Points		Interpretation
Under 35 points	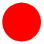	The athlete’s readiness to return to play is not adequate
Between 35 and 39 points	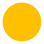	Other complementary tests should be taken into account
More than 40 points	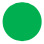	The athlete can return to play with certain assurances
Score obtained		
41 points	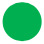	The athlete can return to play with certain assurances

## Data Availability

The raw data supporting the conclusions of this article will be made available by the authors, without undue reservation.

## Supplementary Materials

The following supporting information can be downloaded at: https://www.mdpi.com/article/10.3390/ijerph23010026/s1, File S1: Semi-structured interview; File S2: Assessment of psychological intervention; File S3: Analysis of the return to competition.

## Abbreviations

The following abbreviations are used in this manuscript:

ACLR	Return after ACL reconstruction
ACL	Anterior cruciate ligament
RTP	Return to play
FMPS	Frost Multidimensional Perfectionism Scale
STAI	State-Trait Anxiety Inventory
BDI-II	The Beck Depression Inventory-II
GHQ-12	General Health Questionnaire
PSQI	Pittsburgh Sleep Quality Index
PCS	Pain Catastrophizing Scale
VAS	Visual Analog Scale
POMS	Profile of Mood States
DASS-21	Depression, Anxiety and Stress Scales
PRIA-RS	Psychological Readiness of Injured Athlete to Return to Sport
