# Supplementary material for: Beyond the Injury: A Case Report on Psychological Intervention During ACL Rehabilitation in a Professional Futsal Player
Source: Int J Environ Res Public Health. 2025 Dec 23;23(1):26. doi: 10.3390/ijerph23010026 (PMC12841408; doi:10.3390/ijerph23010026)
Supplement: Supplementary file 1 [file ijerph-23-00026-s001.zip › ijerph-4021141-supplementary/Appendix 1. Semi-structured interview.pdf]

Appendix 1. Semi-structured interview

Analysis of the background to the injury, expectations and psychological state

The player mentions that she used to be a regular starter and was gradually getting more minutes on the pitch: *"I was feeling good. I was finally seeing the positive side of the sport."* She had experienced a break due to injuries and notes that she had a chance of being called up to the national team. On a personal level, she describes herself as a perfectionist in certain areas: *"I worry about what others might think."* She also considers herself to be sociable and, although she doesn't have much free time, she usually goes out with her friends when she can. Futsal plays a significant role in her life. She says she gets on well with her coach, and about her teammates she comments: *"We're like a little family."* In terms of her family life, she shares that her parents are very involved in her recovery process. They struggled during her injury, and she doesn't want to give them any more reasons to worry. Finally, regarding her expectations about the injury and the psychological support, the player believes she will come out mentally stronger and that overcoming this period will be a positive experience: *"I'll start to take things more calmly and dedicate more time to myself, to get to know myself better."* She expresses a strong desire to recover as well as possible, *"to be like I was before the injury,"* and believes that psychological support can help her in all areas of her life.

Analysis of the moment of injury

The injury occurred during a match, in a challenge for the ball. An opposing player pushed her, and as she fell, she felt that "something happened that hadn't happened before." Upon learning the diagnosis, she thought: *"Again, I have to undergo surgery."* The player says she felt a strong sense of anger and spent some time feeling as though she were in a bubble: *"It was hard for me to express what I was feeling."* She also felt disappointed, wondering whether it could have been avoided. She recalls that, upon leaving the pitch, she was crying and shouting. She was taken to a room where some tests were carried out to confirm the injury. After that, she stayed on the bench supporting her teammates. On a more positive note, she mentions that she felt a certain sense of calm due to her previous experience with injuries. She also felt supported by both her teammates and her coach.

As a possible explanation for the injury, she believes that psychologically, the thought of getting injured again may have played a role, as she had been experiencing some discomfort and had suffered previous injuries. Regarding physical preparation, she states that it is something that is worked on extensively and effectively. In her psychological assessment of the moment the injury occurred, the player reported the following levels (1= low; 5 = very high):

- Perceived pressure from the coach: 3 (moderate)
- Personal motivation: 5 (very high)
- Self-demand: 4 (high)
- Focus on the task: 3 (moderate)
- Perceived psychological coping resources: 1 (low)

Assessment of the objective and subjective consequences of the injury

Among the objective consequences, she highlights her dependence on others for getting around and carrying out basic tasks. She feels that her active lifestyle and personal freedom are restricted, as she's unable to go out much. She mentions spending a significant amount of time on rehabilitation and notes the loss of training sessions, matches, and major competitions such as the Super Cup and the University Championship. She also states that she has had to pause her work as a coach.

Regarding the subjective consequences, she says: *"I find it hard to concentrate because I'm always thinking about my knee,"* and *"I overthink the recovery timelines."* She expresses fear that she might not perform at the same level as before and could be left out of the team. Additionally, she comments: *"I wouldn't be surprised if I relapsed,"* reflecting a certain expectation or normalisation of the risk of reinjury.
