# Supplementary material for: Beyond the Injury: A Case Report on Psychological Intervention During ACL Rehabilitation in a Professional Futsal Player
Source: Int J Environ Res Public Health. 2025 Dec 23;23(1):26. doi: 10.3390/ijerph23010026 (PMC12841408; doi:10.3390/ijerph23010026)
Supplement: Supplementary file 1 [file ijerph-23-00026-s001.zip › ijerph-4021141-supplementary/Appendix 2. Assessment of psychological intervention.pdf]

Appendix 2. Assessment of psychological intervention

This questionnaire aims to reflect on the psychological preparation carried out, evaluating different aspects of it. We will use both quantitative and qualitative evaluation, which may help to better assess the effectiveness of techniques, strategies, and procedures.

In general terms (0 = not at all; 10 = very much)

---

1. Are you satisfied with the psychological preparation you received?

9

Indicate the aspects you liked the most and explain why: Visualisation, because it helped me to imagine things I couldn't do and kept me motivated.

Indicate the aspects you liked the least and explain why: If I had to mention something, I would say the attentional focus exercise, due to its difficulty.

---

2. To what extent do you think it has helped you in the rehabilitation of your injury?

10

Indicate the aspects that were most helpful and explain why: The sleep hygiene work, because it helped me develop habits I didn't have before, and as a result, perform with greater capacity.

---

3. To what extent do you think it can help you in your return to sport, both in training and in competitions?

10

---

4. Has the psychological preparation helped you in other areas outside the rehabilitation process?

9

Indicate and explain in which areas the psychological preparation has been helpful: The relaxation and visualisation techniques, because I am a very nervous person and tend to get stressed out quickly over everything.

5. Rate your satisfaction and the effectiveness of the techniques and strategies used

Psychological techniques	Quantitative rating SATISFACTION										
	**0**	**1**	**2**	**3**	**4**	**5**	**6**	**7**	**8**	**9**	**10**
Goal setting										X	
Relaxation techniques											X
Sleep higiene programme											X
A-B-C programme									X		
Visualisation											X

Psychological techniques	Quantitative rating EFFECTIVENESS										
	**0**	**1**	**2**	**3**	**4**	**5**	**6**	**7**	**8**	**9**	**10**
Goal setting											X
Relaxation techniques											X
Sleep higiene programme											X
A-B-C programme										X	
Visualisation											X

**Psychological techniques**	**General qualitative evaluation** *(Indicate what you consider most representative of each technique or strategy; whether you have used it a lot or not, whether it was easy or not, and any other relevant aspect.)*
Goal setting	Guidelines you need to work with. Each phase takes its time. Patience is required.
Relaxation techniques	Widely used and simple.

Sleep higiene programme	Importance of sleep and how it can affect us. Widely used.
A-B-C programme	Simple, although little used.
Visualisation	Complicated but satisfactory.
