# Supplementary material for: Beyond the Injury: A Case Report on Psychological Intervention During ACL Rehabilitation in a Professional Futsal Player
Source: Int J Environ Res Public Health. 2025 Dec 23;23(1):26. doi: 10.3390/ijerph23010026 (PMC12841408; doi:10.3390/ijerph23010026)
Supplement: Supplementary file 1 [file ijerph-23-00026-s001.zip › ijerph-4021141-supplementary/Appendix 3. Analysis of the return to competition.pdf]

Appendix 3. Analysis of the return to competition

Match	Result	Performance Rating (0–10)	Emotional State	Observations
Matchday 4	1–0 (win)	8	Excited, motivated, in flow	Memories from the past. The crowd chanting my name. Very special match. I felt the support.
Cup Match	1–2 (win)	Did not play	Sadness due to not playing	I understand that I need to ease back in gradually.
Matchday 5	2–0 (loss)	3	Wandering. Angry about my performance. Frustrated.	Felt like I wasn't really in the game. Unfocused.
Matchday 6	4–1 (win)	7	Frustrated about not being able to play more	Felt like I could have played more.
Matchday 7	1–4 (win)	8 (scored a goal)	Very motivated	Saw a friend from the opposing team who has supported me throughout the process. A great support.
Cup Match	1–5 (loss)	9 (scored a goal)	Great on a personal level. I felt all kinds of emotions, both positive and negative	It was an important match.
Matchday 8	4–4	7	Focused on enjoying, without feeling pressure	A lot of stimuli, changes in the venue, cameras broadcasting the match.
