# Supplementary material for: Beyond the Injury: A Case Report on Psychological Intervention During ACL Rehabilitation in a Professional Futsal Player
Source: Int J Environ Res Public Health. 2025 Dec 23;23(1):26. doi: 10.3390/ijerph23010026 (PMC12841408; doi:10.3390/ijerph23010026)
Supplement: Supplementary file 1 [file ijerph-23-00026-s001.zip › ijerph-4021141-supplementary/Declaration of Informed Consent.pdf]

DECLARATION OF INFORMED CONSENT

Mr./Ms. ...., aged ….. years[1] and with DNI Nº……..……… , declares that he/she has been informed of the objectives of the study entitled 'Impact of injury frequency and severity on mental health indicators of triathletes: a longitudinal study, and of the techniques to be used to achieve these objectives and the tests for the evaluation of the psychological variables under study.

I have also been informed that my personal data will be protected and included in a file that must be subject to and with the guarantees of the law 15/1999 of 13 December.

I have also been informed that I can withdraw my participation in the study at any time without giving any explanation and without any prejudice.

Taking this into consideration, I GIVE my CONSENT to carry out the psychological assessment and training planned in this project in order to meet the objectives specified in the project.

Date: ……., ………………., ……………..

Signed Mr./Mrs.

[1] In the case of minors, the express informed consent of both parents must be provided in all cases.
